# Chemical Pathology in Its Relation to Practical Medicine

**Published:** 1902-08

**Authors:** 


Chemical Pathology in its Relation to Practical Medicine. By C. A. Herter, M. D., Professor of Pathological Chemistry in the University and Bellevue Medical College, New York, etc. In one 12mo volume of 454 pages. Philadelphia and New York: Lea Brothers & Co. 1902. [Cloth, $1.75 net.]

For many years Dr. Herter has been engaged in researches in chemical pathology and in his published articles in current literature has given some idea of the scope of his work. The present volume is a collection of those papers arranged in the form of lectures and were delivered before the students of the University and Bellevue Hospital Medical College, in New York, the past winter. They show that the instincts of a practical clinician have guided a master hand in the treatment of such topics as: Clinical defences of the organism against disease; Food-stuffs and their fate in the body in health and disease;

Chemical pathology of gastric and intestinal digestion; Chemical pathology of hepatic disease; Diabetes; Starvation; Undernutrition and obesity. Other chapters deal with the iron of the food and its fate in the body, alcohol, the organic acids of the food, tea and coffee, excessive fermentation and putrefaction in the digestive tract.

Dr. Herter is an accepted authority in pathology and chemistry and is thoroughly well equipped for the task of selecting the new and perhaps revolutionary facts discovered in this field of investigation and demonstrating their application and usefulness in the broader realm of daily practice.

The work will prove interesting reading to those who desire to know what nature does in her dietary. It is a work of exceptional value and timely interest.

Lea Brothers & Co. have made the work durable and pleasing to the eye. W. C. K.

